# Natural Selection Fails to Optimize Mutation Rates for Long-Term Adaptation on Rugged Fitness Landscapes

**DOI:** 10.1371/journal.pcbi.1000187

**Published:** 2008-09-26

**Authors:** Jeff Clune, Dusan Misevic, Charles Ofria, Richard E. Lenski, Santiago F. Elena, Rafael Sanjuán

**Affiliations:** 1 Department of Computer Science and Engineering, Michigan State University, East Lansing, Michigan, United States of America; 2 Instituto de Biología Molecular y Celular de Plantas, Consejo Superior de Investigaciones Cientificas, Universidad Politecnica de València, València, Spain; 3 Institute of Integrative Biology, ETH Zurich, Zurich, Switzerland; 4 Department of Microbiology and Molecular Genetics, Michigan State University, East Lansing, Michigan, United States of America; 5 The Santa Fe Institute, Santa Fe, New Mexico, United States of America; 6 Institut Cavanilles de Biodiversitat i Biologia Evolutiva, Universitat de València, València, Spain; University of Bath, United Kingdom

## Abstract

The rate of mutation is central to evolution. Mutations are required for adaptation, yet most mutations with phenotypic effects are deleterious. As a consequence, the mutation rate that maximizes adaptation will be some intermediate value. Here, we used digital organisms to investigate the ability of natural selection to adjust and optimize mutation rates. We assessed the optimal mutation rate by empirically determining what mutation rate produced the highest rate of adaptation. Then, we allowed mutation rates to evolve, and we evaluated the proximity to the optimum. Although we chose conditions favorable for mutation rate optimization, the evolved rates were invariably far below the optimum across a wide range of experimental parameter settings. We hypothesized that the reason that mutation rates evolved to be suboptimal was the ruggedness of fitness landscapes. To test this hypothesis, we created a simplified landscape without any fitness valleys and found that, in such conditions, populations evolved near-optimal mutation rates. In contrast, when fitness valleys were added to this simple landscape, the ability of evolving populations to find the optimal mutation rate was lost. We conclude that rugged fitness landscapes can prevent the evolution of mutation rates that are optimal for long-term adaptation. This finding has important implications for applied evolutionary research in both biological and computational realms.

## Author Summary

Natural selection is shortsighted and therefore does not necessarily drive populations toward improved long-term performance. Some traits may evolve because they provide immediate gains, even though they are less successful in the long run than some alternatives. Here, we use digital organisms to analyze the ability of evolving populations to optimize their mutation rate, a fundamental evolutionary parameter. We show that when the mutation rate is constrained to be high, populations adapt considerably faster over the long term than when the mutation rate is allowed to evolve. By varying the fitness landscape, we show that natural selection tends to reduce the mutation rate on rugged landscapes (but not on smooth ones) so as to avoid the production of harmful mutations, even though this short-term benefit limits adaptation over the long term.

## Introduction

Mutation is the ultimate source of genetic variation, and thus the rate at which spontaneous mutations appear is a fundamental evolutionary parameter. The mechanisms of DNA replication and repair are themselves genetically encoded and variable [1]–[5], making mutation rates potential targets of evolutionary optimization. Two opposing forces contribute to the evolution of mutation rates. On the one hand, most mutations with phenotypic effects are deleterious, producing a genetic load that favors organisms with low mutation rates; on the other hand, beneficial mutations are necessary for adaptation. Given this trade-off between genetic load and adaptation, there should exist an intermediate mutation rate—hereafter referred to as the ‘optimal’ rate, or *U_opt_*—that balances these forces and maximizes adaptation over the long-term [6]–[9]. It is important, however, to note that these two forces operate at different timescales. The costs of genetic load are continuously paid in the short-term, whereas the payoffs of adaptation come in the long-term [6]–[8], [10]–[12].

Experiments have shown that genotypes with increased mutation rates can be favored by selection if they face novel or changing environments [1], [13]–[21]. Similarly, recent work with RNA viruses has shown that certain high-fidelity genotypes have diminished fitness and virulence in mice [22],[23], which might reflect their restricted ability to create the genetic variability needed to escape from immune surveillance. However, another recent study with an RNA virus failed to observe a positive association between mutation rate and the rate of adaptation to a novel environment [24]. Despite their importance, these studies suffer from some unavoidable limitations. For example, it is unknown whether the observed mutation rates are the product of evolutionary optimization or, alternatively, if they are far from their optimal values. Also, it is often difficult to assess whether experimental observations reflect evolutionary equilibria or transient states.

These limitations can be overcome using evolution with digital organisms owing to the speed and ease of data collection. Digital organisms are self-replicating computer programs that inhabit a virtual world where they reproduce, mutate, compete for resources, and evolve according to the same fundamental processes as biological organisms [25]. Here, we use digital organisms to study the ability of natural selection to adjust the mutation rate. We first validate the existence of an optimal mutation rate by extensively exploring a range of mutation rates and observing which rate maximizes adaptation over the long-term. Then we allow mutation rates to evolve under natural selection and assess whether the optimal rate is reached. Even in conditions highly favorable for mutation rate optimization, mutation rates systematically evolve that are far below the optimum, showing that natural selection fails to optimize mutation rates. We propose a novel hypothesis for these results based on the topology of the underlying fitness landscape, and we then proceed to experimentally test it.

## Results

### Selection Fails To Find the Optimal Mutation Rate

**Figure 1:**
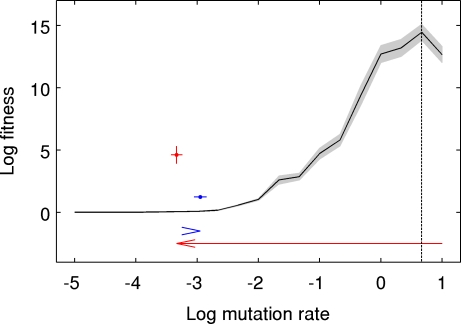
Evolution of suboptimal mutation rates on a complex fitness landscape. Fitness is shown as a function of the genomic mutation rate. The solid line shows mean fitness of the final population, itself averaged over 50 runs, for 15 different static mutation rates (*U* = 10^−5^, 10^−4^ and from 10^−3^ to 10 at 1/3 log_10_ intervals). The shaded area represents±1 s.e.m. The optimal mutation rate—the rate that maximized final fitness—was *U_opt_*≈4.641 (vertical dashed line). The two colored points show the mean fitness and mutation rate of the final population, averaged over 50 runs, in experiments where mutation rates freely evolved with starting values of either 10 (red) or 10^−3^ (blue) (error bars represent±1 s.e.m). Evolved mutation rates and fitness values were both orders of magnitude lower than those observed in the experiment with *U_opt_*.

We studied the evolution of mutation rates using the Avida digital evolution platform [25]–[34]. To test empirically whether there was an intermediate, optimal rate of mutation that maximized adaptation, we performed a series of evolution experiments. In each experiment, a genetically homogenous population was placed in a novel environment where it evolved for 150,000 updates (∼15,000 generations) at a constant mutation rate (see Methods). We explored 15 different mutation rates spanning six orders of magnitude (10^−5^ to 10 mutations per genome per generation). The final fitness values confirmed that there was an optimal mutation rate at an intermediate value, with *U_opt_*≈4.641 (Figure 1). An analysis of the temporal dynamics of these experiments showed that this rate yielded the highest fitness from about generation 230 onward. Interestingly, for the very earliest time points (before generation 50), the lowest mutation rate (10^−5^) produced the highest fitness values, whereas for generations 50–230 a mutation rate of 2.2 gave the highest fitness values.

**Figure 2:**
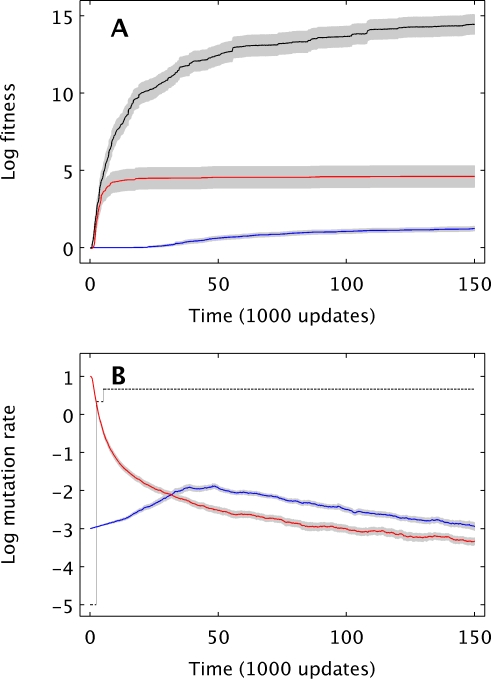
Evolutionary trajectories for fitness and mutation rate on a complex fitness landscape. (A) Evolution of average log-fitness±1 s.e.m. for treatments with the mutation rate fixed at *U_opt_* = 4.641 (black) and for treatments with variable mutation rates starting at either 10 (red) or 10^−3^ (blue). (B) Evolution of average log genomic mutation rate±1 s.e.m. for treatments with variable mutation rates starting at either 10 (red) or 10^−3^ (blue). The black line indicates the mutation rate that had produced the highest average fitness for that time point.

To assess whether evolution would produce organisms with mutation rates near the long-term *U_opt_*, we ran additional experiments in which mutation rates were allowed to change (see Methods), starting from rates either below (10^−3^) or above (10) the optimum. Strikingly, mutation rates evolved to levels far below the long-term *U_opt_*, regardless of the starting value (Figure 1). In light of our observation that the optimum rate can change over time, one might hypothesize that the typical mutation rate of an evolving population had actually followed a near-optimal trajectory throughout its evolution, but that the final mutation rate is not a good indicator of the ability to optimize the mutation rate. However, this explanation can be ruled out because the final average fitness of the populations whose mutation rates could change was significantly lower than the fitness levels of the populations that evolved at a constant *U_opt_*. The log-transformed final fitness values for treatments with changing mutation rates were 4.61±0.70 and 1.23±0.15 (mean±1 s.e.m.) for the populations starting at high and low initial rates, respectively. Both of these values are significantly lower than the 14.45±0.64 obtained for populations evolved at *U_opt_* (Mann-Whitney tests, both *P*<0.001). The fitness advantage for *U_opt_* is also clear for nearly all intermediate time points (Figure 2A). While populations starting below *U_opt_* did experience a transient increase in their mutation rates (Figure 2B), the mutation rates still stayed more than two orders of magnitude below *U_opt_*. For populations starting above *U_opt_*, the results were particularly striking because selection pushed the populations through the optimal rate on their way to an evidently very suboptimal rate (Figures 1 and 2B).

The finding that mutation rates evolved to be suboptimal was robust to diverse and substantial changes in the experimental conditions. First, we tested whether our results depended on the particular ancestral organism used. In the original experiments, the ancestor was a default, hand-coded organism. To assess whether this condition substantively influenced our results, we let a population founded by this organism adapt for 50,000 updates to an environment without any rewarded functions, using *U* = 4.641. The most abundant genotype at the end of this preliminary run was then used as the ancestor in repetitions of our original experiments. Second, we modified the complexity of the environment by varying the number of rewarded functions. Third, we tested the effect of environmental fluctuations by introducing periodic changes in the set of rewarded functions. In some of these experiments the non-rewarded functions were neutral, and in others performing these functions reduced fitness. The rate at which environmental fluctuations occurred was also varied. Fourth, we experimented with different implementations of how mutation rates could themselves change over time. In the original experiments, each organism's mutation rate had a constant probability Π of changing every generation, and the magnitude of any resulting change was controlled by a dispersion parameter *σ*, with Π = 0.5 and *σ* = 0.1. We conducted additional experiments in which we lowered Π, raised *σ*, or both by orders of magnitude. We also explored a configuration where increases in the mutation rate were more likely than decreases, as may happen in biological systems where it is more likely for mutations to harm than to improve an existing DNA repair pathway. Finally, we let the mutation rate apply reflexively to itself, such that high-fidelity genotypes rarely changed their mutation rates whereas low-fidelity genotypes did so frequently. In all of these additional experiments, mutation rates evolved to suboptimal levels (data not shown). We conclude, therefore, that selection fails to optimize mutation rates for long-term adaptation in a broad range of experimental conditions.

### Selection Favors Suboptimal Mutation Rates Because They Are Advantageous in the Short Term

A possible explanation for why mutation rates evolved to be much lower than *U_opt_* is that selection favored those genotypes that minimized the short-term fitness costs caused by deleterious mutations. This explanation is supported by the observations that, during the earliest generations of the evolution experiments, the lowest mutation rate yielded the highest fitness values. To test whether short-term selection would favor low mutation rates, we performed competition experiments between two kinds of organisms, designated A and B. These organisms were identical except for their mutation rate, which was set to *U_opt_* for A and 0 for B; neither mutation rate was allowed to change during the competition. All competitions were conducted with the same environmental configurations as in the main experiments. In all of 50 runs, B drove A extinct in fewer than 40 generations. Competitions were also performed using *U* = 1.0 and *U* = 2.154 for B in order to address whether selection would also favor less extreme reductions in mutation rate. In both treatments, B drove A extinct in all 50 trials in fewer than 800 generations. These experiments confirm our hypothesis that natural selection was shortsighted and favored low mutation rates, even when such low rates precluded further adaptation.

### Whether an Optimal Mutation Rate Can Evolve Depends on the Ruggedness of the Fitness Landscape

**Figure 3:**
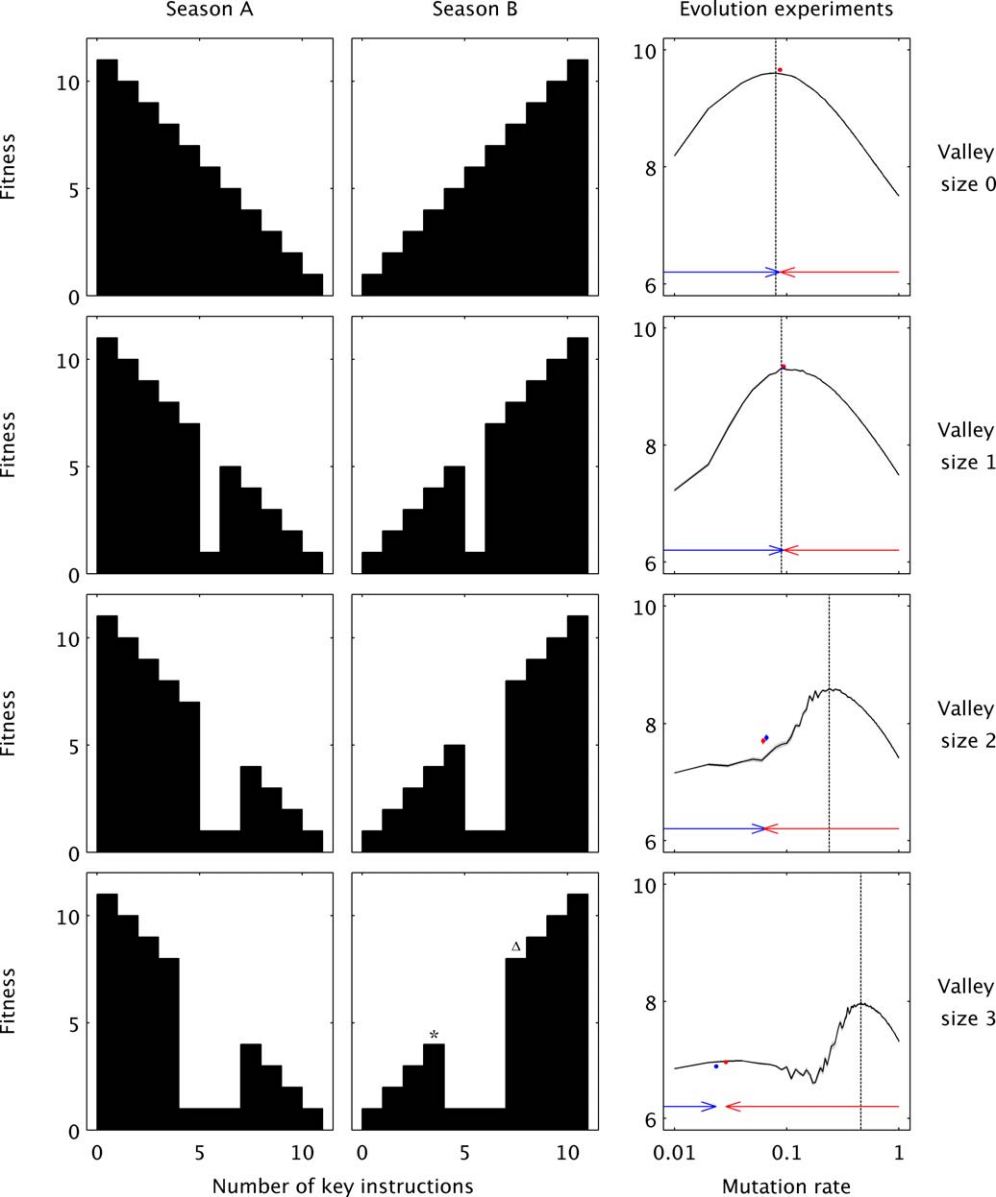
Evolution of mutation rates on simple fitness landscapes with different ruggedness. Here, fitness depended solely on the match between the environment and the number of a key instruction that organisms had in their genomes. In season A (left column) the key instruction was deleterious while it was beneficial in season B (center column). Rugged fitness landscapes with maladaptive valleys (rows 2–4) were introduced by setting the fitness of organisms with intermediate numbers of the key instruction to the minimum fitness level of one. The right-most column shows the results of evolution experiments under each of these selective regimes. Final fitness is shown as a function of genomic mutation rate for both static and dynamic mutation rates. The solid black line represents the average of the mean fitness across 10 runs for each of 100 different static mutation rates ranging from *U* = 0.01 to 1 in increments of 0.01. The two colored points represent the mean fitness and mutation rate, both averaged over 50 runs where the mutation rate freely evolved, with initial rates of *U* = 1 (red) or 10^−5^ (blue). Mutation rate and fitness values were time-averaged over the last 10 of 50 environmental changes. Owing to very similar final values, despite the very large initial differences, the individual colored points are indistinguishable in the first two rows, and error bars are not visible. The arrows indicate where mutation rates began and ended, on average, for the dynamic-rate experiments. Although the optimal mutation rate increases as a function of valley size (note the right-shift in the dashed line from top to bottom), the evolved mutation rates in fact decrease as a function of valley size (note the left-shift of the blue and red points from top to bottom).

We conclude from the results presented thus far that the failure of the evolving populations to achieve or even maintain the mutation rates that maximize long-term adaptation reflect the conflict between the short-term cost of deleterious mutations and the long-term potential for adaptive evolution. We further hypothesize that the resolution of this tension may depend on the topology of the fitness landscape on which evolution occurs. In a rugged fitness landscape, where there are multiple peaks separated by maladaptive valleys [35],[36], populations at a local optimum must traverse regions of low fitness in the short-term in order to reach higher-fitness solutions in the long-term. This conflict leads us to hypothesize that the inability of natural selection to optimize mutation rates may depend on the ruggedness of the fitness landscape. The ideal test of this hypothesis requires comparing the evolution of mutation rates on fitness landscapes with and without fitness valleys. This test cannot be performed using the standard Avida setup, owing to the presence of extensive genetic interactions that make the fitness landscape complex and rugged [23]. We therefore modified Avida to allow simple, explicit, user-defined fitness functions that allowed us to manipulate the ruggedness of the fitness landscape (Methods, Figure 3). Adaptation occurs so fast when using these simple configurations that we also had to make the environment fluctuate between two ‘seasons’ in order to ensure a continual opportunity for beneficial mutations. These fluctuations mean that genotypes that are more fit in one season are less fit in the other (Figure 3).

A quantitative investigation of mutation rates spanning orders of magnitude revealed, once again, that intermediate mutation rates were optimal over the long-term (Figure 3). We then allowed mutation rates to evolve starting at a genomic mutation rate either below (10^−5^) or above (1) the long-term optimum. Near-optimal values were efficiently selected in those landscapes without a fitness valley or with a narrow valley (Figure 3, rows 1 and 2). However, as the width of the valley grew, mutation rates evolved to be orders of magnitude lower than *U_opt_* (Figure 3, rows 3 and 4). Fitness values were again used to judge the optimality of mutation rates. With no valleys or with narrow valleys, the average fitness in populations with variable mutation rates was slightly above that of populations with a constant rate of *U_opt_* (Figure 3, rows 1 and 2, Mann-Whitney test, *P*<0.001 in both cases), which indicates a small benefit of adjusting mutation rates during evolution [37]. In stark contrast, for wider valleys, the average fitness in populations with variable mutation rates was far below that of populations with a constant rate of *U_opt_* (Figure 3, , rows 3 and 4 Mann-Whitney test, *P*<0.001 in both cases), confirming that the evolved mutation rates were suboptimal on the these rugged landscapes.

**Figure 4:**
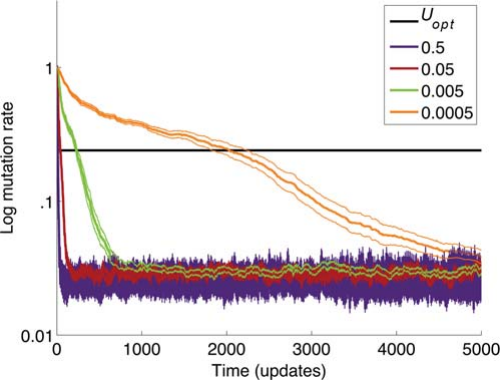
Evolutionarily stable mutation rate does not depend on the frequency with which the mutation rate changes (Π). The evolution of mutation rates in the explicit fitness landscape with a valley size of three is shown for several values of Π, as indicated by the colored key. Each curve shows the average of 20 runs; the adjacent bands represent±1 s.e.m. The value of *U_opt_* was determined in previous experiments (see text). The rate of approach toward the evolutionarily stable mutation rate depends on Π, but the equilibrium value itself does not.

These results show that there exists a conflict between short-term and long-term evolutionary strategies on rugged landscapes. In the short-term, low mutation rates are favored because they reduce the load of deleterious mutations, whereas in the long-term, high rates are favored because they increase the chance of producing beneficial mutants. Whether the short-term interests dominate, allowing genotypes with suboptimal mutation rates to spread, should be a function of the expected waiting time until the discovery of a beneficial mutant. To test this prediction, we competed genotypes with either optimal or suboptimal rates in the explicit fitness landscape with a valley size of three (Figure 3). In one set of experiments, we placed all organisms of both types on the low local fitness peak (asterisk in Figure 3) and let them compete for 300 generations (the duration of one season in the previous experiments). We then repeated the same experiments except that one of the individuals with the long-term optimal mutation rate started on the other side of the valley (triangle in Figure 3), such that the waiting time for the production of a beneficial mutant was eliminated. A comparison between these two sets of competition experiments shows that the probability that a genotype with a mutation rate that is below the long-term optimum can invade declines significantly when the waiting time to discover beneficial mutants is artificially eliminated (Table 1). This result illustrates why wider valleys, which create longer waiting times for beneficial mutants, cause the evolution of suboptimal mutation rates.

**Table 1:** Outcomes of competitions between lineages with optimal (*U_opt_* = 0.24) versus suboptimal (*U_subopt_*) mutation rates in the explicit fitness landscape with a valley size of 3.

	With Waiting Time			Without Waiting Time			
*U_subopt_*	*U_opt_* Fixed	*U_subopt_* Fixed	Neither Fixed	*U_opt_* Fixed	*U_subopt_* Fixed	Neither Fixed	*P*
0	238	2	10	249	0	1	0.0082
0.06	149	24	77	242	0	8	<0.0001
0.12	185	12	53	229	1	20	<0.0001

A total of 250 runs were performed for each treatment shown below. The two lineages started with equal numbers in all cases. The entries show the number of times that each lineage was fixed (i.e., reached 100% of the total population) or that neither lineage was fixed within 300 generations. With waiting time: all individuals started at the lower fitness peak (asterisk in Figure 3). Without waiting time: one individual belonging to the lineage with *U_opt_* started on the other side of the fitness valley (triangle in Figure 3). Three different values of *U_subopt_* were examined. *P* values are based on χ^2^ tests (with 2 degrees of freedom) that measured the effect of waiting time.

The reader may also notice that the probability of invasion by the genotype with the suboptimal mutation rate was rather small in both sets of experiments (Table 1). This observation might seem, at first glance, to be at odds with the fact that mutation rates evolved over the long run to be extremely suboptimal (Figure 3, rows 3 and 4). This difference makes sense, however, for two interrelated reasons. First, each environmental change that follows the fixation of a mutation on one adaptive peak requires another waiting period for a beneficial mutation, which provides another opportunity for invasion by a genotype with a suboptimal mutation rate that reduces the mutational load. Second, any reductions in the mutation rate become self-reinforcing, as the lower mutation rates make it less likely to generate a beneficial mutant on a distant peak, which increases the expected waiting time for the generation of the next beneficial mutants, thereby increasing the opportunity for a genotype with an even lower mutation rate to invade.

Finally, we examined whether the frequency with which the mutation rate changes (in essence, the mutation rate in the pathway that encodes the mutation rate), which we call Π, affects the evolutionarily stable mutation rate. Our intuition was that lower values of Π would make contests between lineages with different mutation rates less frequent, but that the long-term results of many such contests would remain the same. To test this prediction we again used the explicit landscape with a valley size of three. Even when Π varied over four orders of magnitude, it did not affect the final mutation rate that was reached (Figure 4). Hence, the inability of selection to optimize the mutation rate for long-term adaptation depends on the topology of the fitness landscape, but not on the frequency with which the mutation rate itself changes.

## Discussion

We have shown that mutation rates evolve to near-optimal levels on extremely smooth fitness landscapes. However, if fitness landscapes are rugged, and the maladapted valleys between nearby fitness peaks are wide, then the scarcity of immediately accessible beneficial mutations tips the scale such that short-term selection favors mutation rates that are far below the optimum that would produce the fastest long-term adaptation. Moreover, this process is self-reinforcing because the lower the mutation rate, the less likely it becomes to produce a genotype on the other side of the fitness valley, thereby effectively widening the valley. The digital organisms in the standard Avida configuration used in our first set of experiments exhibit extensive and variable genetic interactions, making the fitness landscape rugged [23]. In those experiments, populations invariably evolved to have mutation rates that were far below the rate that would maximize their long-term fitness gains. We hypothesized that the ruggedness of the landscape was responsible for this inability to optimize their mutation rate for long-term adaptation. In order to test this hypothesis rigorously, we had to change the fitness landscape in Avida from one that is an emergent feature of complex interactions among many instructions to a much simpler surface that could be tuned to be either smooth or rugged. We found that evolving populations were indeed able to achieve mutation rates that maximized their rate of adaptation on smooth landscapes, whereas they became stuck at much lower mutation rates when the valleys between fitness peaks became too large, thus confirming our hypothesis. A growing body of experiments with viruses, bacteria, yeast, and higher eukaryotes shows that epistatic interactions are widespread and vary in their sign and intensity, implying that natural fitness landscapes are also often rugged [35],[36],[38]. Thus, our finding that rugged fitness landscapes can impede the optimization of mutation rates for long-term evolutionary adaptation is relevant to the natural world.

Our experiments were performed under conditions that were favorable for the optimization of mutation rates. First, the organisms reproduced asexually. Both theoretical [12],[39],[40] and experimental work [15] has shown that asexuality facilitates the evolution of elevated mutation rates, because sexual recombination breaks up the linkage between mutator alleles that increase mutation rates and the beneficial mutations that are generated by the mutators. Second, to ensure that beneficial mutations were always available, our experiments used either an environment with more rewarded functions than the organisms ever evolved during a run (standard configuration) or a changing environment (explicit landscapes configuration). Third, population sizes were large and strong directional selection was imposed, so that drift was only a minor force in our experiments. Smaller populations might traverse maladaptive valleys more easily, owing to increased drift. However, small populations would be less likely to generate the multiple simultaneous mutations that would allow them to leap across these valleys in a single generation. In populations much larger than those we tested, the probability of an adaptive leap involving multiple simultaneous mutations would increase, but selection should be more powerful in preventing a multi-generation transition across a valley via drift. The effect of population size on the optimal mutation rate, and on the evolution of suboptimal mutation rates, thus remains an interesting area for future investigation. Nevertheless, while the optimal mutation rate and the precise width of the valley that is necessary to cause the evolution of a suboptimal rate may depend on population size, we would not expect that dependency to undermine the general conclusion of this paper, namely, that on sufficiently rugged fitness landscapes, mutation rates will evolve to be suboptimal for long-term adaptation.

The inability of evolving populations to optimize their mutation rates for long-term adaptation, even with such favorable conditions, indicates that mutation rates will be suboptimal under a wide range of circumstances, at least when fitness landscapes are rugged and populations are far from a global fitness peak. While novel environments can promote increases in the mutation rate if many beneficial mutations become accessible [1], [13]–[21],[40], our work suggests that this rise will be temporary and, moreover, that even the elevated mutation rates may be suboptimal (Figure 2B). Also, given the difficulty of optimizing mutation rates that we have shown, it seems unlikely that stably high mutation rates, such as those for RNA viruses, are maintained primarily because of the rapid adaptive capacity they bestow, as has sometimes been argued [23],[41]. Alternative explanations are needed. For example, the evolution of mutation rates is also influenced by the costs of replication fidelity [8],[23], and recent work has suggested that this cost might explain the high mutation rates observed in RNA viruses [24],[42]. We expect that a cost of replication fidelity, all else being equal, will increase the evolved mutation rate. However, we would not expect the resulting increase to cause the optimization of mutation rates in general, although in a few fortuitous situations the cost of fidelity might increase the evolved mutation rate by just enough to push it near the optimal rate.

Recent theoretical work by Gerrish et al. [43] has predicted that, contrary to our results, natural selection could favor a self-reinforcing increase in mutation rates in asexual populations. This process would continue even until a population suffered a mutational meltdown and went extinct, because a genotype with an increased mutation rate generates greater numbers of deleterious as well as beneficial mutations. Although not explicitly stated, the prediction of Gerrish et al. [43] of a run-away process toward higher mutation rates appears to assume a smooth fitness landscape. However, as we have shown here, the mutation rate typically evolves to a low value on a rugged fitness landscape, so that the runaway process explored by Gerrish et al. should not occur on such landscapes.

Beyond their implications for understanding nature, our findings are also relevant for applied fields that use evolution to improve the performance of biological and computational systems, from molecular and microbial engineering to robotics and evolutionary computation [44],[45]. Researchers using evolution in computational fields have long sought to use natural selection to adjust mutation rates automatically and “on the fly”, in such a way that would sustain and even optimize long-term adaptation [46]–[48]. These efforts were successful on simple “toy” problems [46], but became frustrated when applied to more complex problems because self-adaptive mutation rates generally evolved to suboptimal levels [47],[48]. Our results suggest an explanation: the toy problems had smooth fitness landscapes, whereas the complex problems had rugged landscapes with wide valleys that favored evolutionary conservatism. Our findings also imply that high, fixed mutation rates will often outperform self-adaptive rates on more complex problems, although what the fixed rate should be will depend on the particular problem at hand.

In summary, natural selection is not universally effective at optimizing mutation rates for long-term adaptation; in fact, it is very poor in this respect for populations that evolve on complex fitness landscapes. Also, our results caution against making generalizations based on analyses of simple fitness landscapes, whether one is studying natural systems or using evolution for engineering. As we have shown, the mere inclusion of fitness valleys—which are presumably common to the vast majority of fitness landscapes—can yield radically different conclusions from those based on smooth fitness landscapes.

## Methods

### Experiment One: Standard Configuration

A general description of the Avida software can be found elsewhere [25]. Here, each experiment started with 3,600 identical digital organisms. Genome length was held constant at 100 instructions, with 26 possible instructions per site [27]. Reproduction was asexual. To replicate, an organism first had to copy its genome line by line by repeatedly executing the copy instruction; it then had to execute a divide instruction, which took the offspring and used it to replace a random organism from the population.

During replication, each genomic instruction could mutate to another with probability *μ*, the genomic mutation rate being *U* = 100×*μ*. All instructions were equally likely to result from any given mutation. The mutation rate was held constant in some experiments, while in others the rate could change by evolving over time. In treatments where the mutation rate could change, *μ* had a constant and high probability Π of changing by a small amount during any replication cycle. The magnitude of any resulting change was obtained by drawing log_2_(*μ*_offspring_/*μ*_parent_) values from a Gaussian distribution (0,*σ* ^2^). For the experiments in which mutation rates were more likely to increase than to decrease, we drew log_2_(*μ*_offspring_/*μ*_parent_) from a Gaussian (*bσ* ^2^,*σ* ^2^), where *b* controls the upward bias, and tested values such that mutation rates were up to ∼1.6 times more likely to increase than decrease (though seemingly small, this bias has a large cumulative effect over many generations).

Organisms died when another organism's offspring replaced them or when they executed 2,000 instructions without producing an offspring of their own. All experiments using the standard configuration lasted 150,000 updates. Updates are an arbitrary unit of time in Avida; they represent the time during which each organism, on average, executes 30 instructions [25]. In this configuration, an update corresponded to roughly 0.1 generations, although the precise generation time varied depending on the complexity of the evolved organisms' phenotypes.

Each organism's phenotype depended on the complex rules that governed how its genomic program was executed, and its fitness depended on the interaction between the resulting phenotype and its environment [25]. More specifically, each organism had a metabolic rate that affected how fast it executed instructions, which, in turn, affected its reproduction rate. The ancestral rate doubled with every rewarded logic function that an organism performed. The ancestral organisms could self-replicate but not perform any other function. The ability to perform logic functions evolved by mutation and selection during each run. An organism's fitness, therefore, represents its expected growth rate relative to others in the population and depended on both its replication efficiency and its ability to perform computations. All fitness values are expressed relative to the ancestor. In reporting fitness data, relative fitness values were first averaged over all organisms in a population, then log_10_ transformed, and finally averaged over all replicate populations (independent trials) in an experimental treatment.

To perform logic functions, organisms used inputs consisting of three randomly generated 32-bit strings, which they manipulated to produce an output. The manipulation of these numbers occurred as organisms moved them on and off stacks or between registers by executing instructions such as push, pop, add (combines the numbers in the two specified registers and places the result in a third), shift-r (bit shift right), and so on. A function was rewarded only if the input to output conversion conformed to one of the 77 canonical one-, two- or three-input logic operations. For example, the two-input EQU (‘equals’) function requires inputting two strings and outputting a third string that had a 1 for each of the 32 bits where both inputs had the same value and a 0 where they differed.

Avida runs are inherently stochastic with respect to mutation and death. Therefore, we performed 50 replicate runs for each treatment. Those replicates had identical initial conditions except for a random number seed. That seed affects the outcome of all subsequent stochastic events.

### Experiment Two: Explicit Landscapes Configuration

The standard and the explicit Avida configurations differed in the instruction set, the fitness calculation and the mode of replication. We modified Avida to mimic a two-allele, 10-locus bit-string model used in a previous study [49]. Genome length was always 10, while each “instruction” was either A or B; the ancestral genome was entirely A. Fitness depended only on the number of A or B instructions in an organism's genome, according to the seasonal scheme shown in Figure 3. Every 300 generations the environment fluctuated between the two seasons, and the experiments ran for 15,000 generations. We found empirically that fluctuating the environment more or less frequently than every 300 generations produced smaller fitness differences between the optimal fixed mutation rate and suboptimal mutation rates (data not shown). That high mutation rates are most fit at an intermediate rate of environmental change has been previously shown [49].

In the standard configuration, digital organisms had to copy their genomic instructions in order to replicate, and their fitness depended on their speed of replication as well as any rewards they obtained for performing computational functions. Under this alternative configuration, the organisms did not copy themselves, and only the number of A or B instructions mattered to their fitness. The rest of the setup, such as population size, was identical to the standard configuration.

### Software

All experiments were performed with the Avida software, which can be downloaded for free at http://devolab.cse.msu.edu/software/avida. Default settings were used unless otherwise indicated.
